# Uptake of the Hepatitis B Vaccine Among Pre‐Service Health Professionals in Rwanda

**DOI:** 10.1002/puh2.70019

**Published:** 2024-12-28

**Authors:** Bivegete Kenny Ntwari, Felix K. Rubuga, Silene Jolie Uwingabiye, Vincent Dushimimana, Jean Baptiste Hategekimana, Serieux Cyubahiro, Ivan Steve Rwema, Daniel Ukwishatse, Patrick Karakwende, Jean Muhire, Adolphe Ndikubwimana, Theoneste Ntakirutimana, Pierre Dukuziyaturemye, Didas Mugisha, Canisius Gasana, Edith Musabwa, Felicien Irafasha, Celestin Banamwana, Frank Gasana, Idrissa Nkurunziza, Deborah Oluwaseun Shomuyiwa, Don Eliseo Lucero‐Prisno

**Affiliations:** ^1^ Department of Environmental Health Sciences College of Medicine and Health Sciences School of Public Health University of Rwanda Kigali Rwanda; ^2^ Center for Impact, Innovation and Capacity building for Health Information systems and Nutrition (CIIC‐HIN) Kigali Rwanda; ^3^ Faculty of Pharmacy University of Lagos Lagos Nigeria; ^4^ Department of Global Health and Development London School of Hygiene and Tropical Medicine London UK

**Keywords:** Hepatitis B vaccine, occupational exposure, pre‐service health professionals, uptake, viral hepatitis

## Abstract

**Background:**

High hepatitis B vaccine uptake has led to significant reductions in hepatitis B infection rates and associated health burdens in many countries. Despite the administration of the same vaccine, there has been a lack of emphasis on pre‐service health professionals. This study aimed at assessing uptake of hepatitis B vaccine among pre‐service health professionals at the University of Rwanda.

**Methods:**

This was a cross‐sectional descriptive study. Data were collected using a self‐administered questionnaire, whereas data analysis was performed using SPSS (Version 25; IBM Corp).

**Results:**

A total of 360 respondents participated in the study; among them, 218 (60.6%) were males. About half of the participants, 170 (47.2%), scored between 40% and 60% on the knowledge assessment, whereas the majority of the respondents, 354 (98.3%), were aware of the hepatitis B vaccine. Most of the participants 334 (92.8%), were vaccinated, whereas 231 (69.2%) received the complete 3‐dose vaccination. The most commonly cited reason for not getting vaccinated was lack of awareness (45%). The factors that influenced vaccination status were free vaccination provided by institutions, awareness of the vaccine, and knowledge of hepatitis B infection and its vaccine.

**Conclusion:**

Pre‐service health professionals are at risk of hepatitis infection due to low coverage of hepatitis B vaccination and lack of comprehensive knowledge and awareness regarding the hepatitis B infection and its vaccination.

## Graphical abstract

Although the majority of the participants have been vaccinated with 3 doses (231, 69.2%), pre‐service health professionals in University of Rwanda are occupationally at risk of contracting hepatitis B infection due to low level of fully vaccinated and limited knowledge and awareness regarding HBV infection and its vaccine.

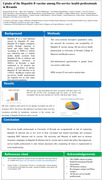

## 1 Background

The emerging viral hepatitis constitutes a public health threat worldwide. Healthcare providers and pre‐service health professionals are exposed double to quadruple times than general population [1]. The risk increases due to accidents of occupational exposure to blood and other biological fluids during treatment of hepatitis B infected people [2, 3]. The risk is high among pre‐service health professionals than experienced healthcare workers due to lack of experience, insufficient training, limited knowledge on how to handle certain instruments, plain carelessness, and fear of reporting occupational exposure, among others. According to World Health Organization (WHO), an estimated 40%–65% of hepatitis B and hepatitis C infections in healthcare workers are attributable to percutaneous occupational exposure especially due to sharp injuries [4].

In 2022, an estimated 254 million people were affected with chronic hepatitis B globally, with 1.2 million new infections each year. During the same year, an estimated 1.1 million deaths occurred from hepatitis B due to cirrhosis and hepatocellular carcinoma [5]. According to WHO, burden of hepatitis B in African region accounts 81 million people who are chronically infected among which 6.1% were adults [6].

Under the global health sector strategy on viral hepatitis 2016–2020, WHO aimed to eliminate viral hepatitis by 2030 that is defined as decrease in number of new cases by 65% and viral hepatitis mortality by 90% in comparison to 2015 situation as baseline [7]. Among components of roadmap toward elimination of viral hepatitis, there is vaccination as key to prevention, which can provide 20 years or lifelong protection that requires no boosters for those who completed vaccination with three shots.

In Rwanda, according to the study conducted in 24 districts out of 30, chronic hepatitis B prevalence was found to be 3.9% in general population [12], whereas a recent study conducted countrywide showed prevalence of chronic hepatitis B to be 2% [13]. A study conducted among healthcare providers in one tertiary hospital found prevalence of hepatitis B virus to be 2.9% [8].

In Rwanda, there are two ways in which hepatitis B vaccine is administered to ensure high level of protection of population against hepatitis B infection and to move forward in global journey toward elimination of viral hepatitis by 2030. First, there is routine birth‐dose vaccination that started since 2002 to be administered to newborns as part of Extended Program of Immunization (EPI) where first shot is administered within first 24 h of birth followed by three shots; second is given in Week 6 after birth, third in 10th week, and last one in 14th week [8]. Second, routine adult vaccination against hepatitis B virus was introduced in 2017 as a catch‐up program to vaccinate all individuals born before 2002 starting from people with high risk of being infected with hepatitis B like healthcare workers [8, 9].

Among the greatest achievement that the Government of Rwanda has made in regard to hepatitis B prevention is vaccination of all individuals in public health system in 2017 and checking for vaccination status of pre‐service health professionals prior to starting clinical work as they are constantly at risk of contracting nosocomial hepatitis B because of their daily contact with patients’ blood and other bodily fluids [10]. In Rwanda, study conducted among healthcare workers in one tertiary hospital found prevalence of hepatitis B to be 2.9%. Another study conducted among nursing students in selected campus that teaches nursing in Rwanda found that 91% have ever received vaccine where only 52% were fully vaccinated with three doses [11] signifying that pre‐service health professionals are at risk due to low vaccine rate.

Although the progress in treatment of hepatitis B virus positive has been made, only small proportion of people have a sustained cure response. Hepatitis B vaccination is the only safe and effective method to reduce incidence and burden of hepatitis B virus and its complications exerted on global health system particularly to high‐risk groups like healthcare providers [12]. Despite the safe and highly effective vaccine, WHO has estimated that hepatitis B virus vaccination coverage among healthcare workers is only 18%–39% in low and middle‐income countries [13]. It is suspected that this is due to the fact that they are not aware of the effectiveness of the vaccine. This study assessed the awareness and uptake of hepatitis B vaccine in pre‐service health professionals.

## 2 Methods

### 2.1 Study Design and Area

A cross‐sectional descriptive design was used to assess the awareness and uptake of hepatitis B vaccine among pre‐service health professionals in University of Rwanda. This research was conducted in three campuses of the University of Rwanda, namely, Remera campus located in Kigali city, Rwamagana campus located in Eastern Province, and Huye campus located in Southern Province.

### 2.2 Study Population and Sampling

Sample size was calculated from 3681 pre‐service health professionals enrolled in University of Rwanda, 2021–2022, College of Medicine and Health Sciences in Remera, Huye, and Rwamagana Campuses. The sample size was calculated for proportions. The final study sample was 361 pre‐service health professionals.

The study utilized the equal probability selection method to select a proportional sample of participants from each stratum (campus) with equal representation in the study. We selected a proportional sample of participants from each stratum (campus), and every student from each campus had equal chance of being a participant of the study.

### 2.3 Inclusion and Exclusion Criteria

The study included undergraduate students in University of Rwanda enrolled in College of Medicine and Health Sciences in Remera, Huye, and Rwamagana Campuses in 2021–2022 academic year and excluded undergraduate students who were unwilling to give consent.

### 2.4 Data Collection Methods and Procedures

The data were collected using questionnaire and were shared to study participants using Google Forms via their emails. Questionnaire was structured into four main sections, first for socio‐demographic information, second for awareness and knowledge regarding hepatitis B infection and its vaccine, third about vaccination status, and the last one about factors influencing hepatitis B vaccination among pre‐service health professionals.

### 2.5 Data Analysis

Data from Google Forms were exported into Excel sheet to be cleaned, and then they were exported to the Statistical Product and Service Solutions Version 25 (SPSS) software for analysis.

Descriptive analysis was performed to generate frequencies and percentages. Chi‐square tests and ANOVA analysis were used for describing the comparison between variables (*p* value <0.05 were declared as statistically significant).

## 3 Results

There were 360 respondents to this study. The details of the study participants are shown in Table 1.

**TABLE 1:** Socio‐demographic characteristics of the study participants.

Characteristics	Frequency (*n*)	Percentage
Age		
18–20	34	9.4
21–25	277	76.9
Above 25	49	13.6
Gender		
Male	218	60.6
Female	142	39.4
Campus		
Huye	176	48.9
Remera	136	37.8
Rwamagana	48	13.3
Department		
Mental health nursing	1	0.3
Ophthalmology	8	2.2
Prosthetic and orthopedic technology	1	0.3
General nursing (bachelor)	48	13.3
Nursing (advanced diploma)	21	5.8
Midwifery	10	2.8
General medicine and surgery	71	19.7
Anesthesia	10	2.8
Biomedical laboratory sciences	31	8.6
Clinical medicine and community health	13	3.6
Clinical psychology	8	2.2
Human nutrition and dietetics	29	8.1
Environmental health sciences	27	7.5
Occupational therapy	1	0.3
Pharmacy	32	8.9
Physiotherapy	16	4.4
Dental therapy	8	2.2
Dental surgery	19	5.3
Medical imaging	6	1.7
Year of study		
First year	92	25.6
Second year	54	15.0
Third year	88	24.4
Fourth year	83	23.1
Fifth year	43	11.9
Residence		
On campus	219	60.8
Off campus	141	39.2
Total	360	100.0

*Note:* Hepatitis B vaccine among pre‐service health professionals in Rwanda 2023.

### 3.1 Awareness on Hepatitis B Vaccine

A high proportion of respondents, 354 (98.3%), were aware of the hepatitis B vaccine compared to their counterparts, 6 (1.7%), who were not aware. Nearly half, 168 (46.7%), reported that they did not participate in any training or continuous education related to hepatitis B vaccine in the past 12 months. The details of assessment on awareness about hepatitis B are shown in Table 2.

**TABLE 2:** Results on awareness assessment.

Item	Frequency (*n*)	Percentage
Awareness on hepatitis B		
Yes	354	98.3
No	6	1.7
Total	360	100.0
More awareness is needed in pre‐service health professionals		
Strongly agree	193	53.6
Agree	138	38.3
Neutral	18	5.0
Disagree	8	2.2
Strongly disagree	3	0.8
Total	360	100.0
Participation in hepatitis B training in last 12 months		
Yes	137	38.1
No	168	46.7
I don't know	55	15.3
Total	360	100.0
Sources of information on hepatitis B		
Television	23	6.4
Newspaper	20	5.6
Social media	77	21.4
Medical training	240	66.7
Total	360	100.0

*Note:* Hepatitis B vaccine among pre‐service health professionals in Rwanda 2023.

Most of the total participants, 334 (92.8%), were vaccinated, as shown in Table 3.

**TABLE 3:** Summary of vaccination status.

Item	Frequency (*n*)	Percentage
Vaccination status		
Yes	334	92.8
No	20	5.6
Maybe	4	1.1
I don't know	2	0.6
Total	360	100.0
Number of doses taken		
1 dose	14	4.2
2 doses	86	25.7
3 doses	231	69.2
More than 3 doses	3	0.9
Total	334	100.0
Reasons for not taking the vaccine		
Lack of awareness	9	45.0
Fear of needle	1	5.0
Too expensive	4	20.0
I don’ t feel the need	5	25.0
It's against my belief or my religion	1	5.0
Total	20	100.0

*Note:* Hepatitis B vaccine among pre‐service health professionals in Rwanda 2023.

Although determining if there was a statistically significant difference between awareness of the hepatitis B vaccine and vaccination status, the significance value was less than 0.001 (*p* < 0.001), indicating a statistically significant difference between being aware of the hepatitis B vaccine and not being aware of it with respect to their influence on hepatitis B vaccination status.

## 4 Discussion

Hepatitis B virus infection has been global public health threat for so long. Healthcare providers as well as pre‐service health professionals in middle‐income countries are particularly at risk of contracting hepatitis B infection. In Rwanda, there is paucity of data regarding knowledge, awareness, and acceptability of hepatitis B vaccine among pre‐service health professionals as at‐risk group to hepatitis B infection. Therefore, current study attempted to assess awareness and acceptability of hepatitis B vaccine among pre‐service health professionals.

According to the results of this study, a large proportion, 98.3%, of the participants were aware of hepatitis B vaccine, and this was almost similar to the results of study conducted in Kenya where 97.9% of the participants were aware [14]. Our study reported higher awareness rates compared to a previous study conducted in Ethiopia among medical and health sciences students, where the awareness rate was 83.7% [15]. Regarding overall knowledge in the present study, only 3.1% scored 80%, and this is in contrast with the study conducted in Nigeria titled “Hepatitis B Knowledge and Preventive Practices Amongst Medical Students in a Tertiary Institution in Abia State, South‐East Nigeria.” [16] where 75.7% of the participants had good knowledge equivalent to the score above 70%. Results from another study in Ethiopia found that 73.6% of medical and health sciences students had good knowledge [15]. Additionally, nearly half of the respondents, 47.2%, scored between 40% and 60% that is classified as poor in many studies. This is in agreement with study titled “Assessment of knowledge and practice towards hepatitis B among medical and health science students in Haramaya University, Ethiopia.” Where 43.8% were within poor knowledge category [17]. Most of respondents knew that hepatitis B infection is transmitted through contact with infected blood and body fluids, needle sharing, and from mother to child. This result was in agreement with the prior findings of Ethiopian study that reported good knowledge on transmission mode and risk factors on hepatitis B infection [18]. Results of a study carried out in Cameroon found that level of knowledge in terms of hepatitis B infection transmission was good [19].

The study found that majority, 92.8%, of the participants were vaccinated against hepatitis B infection, and 69.2% of them were fully vaccinated with three doses. This is almost similar to the study conducted in Kanchipuram where full vaccination rate is 72.5% [20]. However, it is different from a study in Cameroon where only 18% were fully vaccinated [21]. Regarding effectiveness of hepatitis B vaccine in this study, it was found that 61.4% of the respondents reported that vaccine is safe and effective way to prevent hepatitis B infection. This conforms to a study in Nigeria that assessed knowledge among healthcare workers where 98.4% of the respondents signified that vaccine is effective against hepatitis B infection [22].

## 5 Recommendation

The university of Rwanda, Ministry of Health, and Students have to increase awareness campaigns on hepatitis B infection and its vaccine, to ensure that policy requesting pre‐service health professionals to start clinical placement after completing all doses is implemented in every health facility and to make sure that they are fully vaccinated before they enter clinical attachments, respectively.

## 6 Conclusion

The study revealed that about half of the participants, 170 (47.2%), scored between 40% and 60% on the knowledge assessment and only 3.1% scored 80% that indicates participants with good knowledge. Therefore, pre‐service health professionals in University of Rwanda are occupationally at risk of contracting hepatitis B infection due to low level of fully vaccinated and limited knowledge as well as awareness regarding hepatitis B infection and its vaccine.

## Author Contributions

**Bivegete Kenny Ntwari**: conceptualization, investigation, writing–original draft, writing–review and editing, formal analysis, methodology, data curation, software. **Silene Jolie Uwingabiye**: conceptualization, investigation, writing–original draft, writing–review and editing, software. **Vincent Dushimimana**: conceptualization, investigation, writing–original draft, writing–review and editing, formal analysis. **Jean Baptiste Hategekimana**: writing–original draft, writing–review and editing, formal analysis, software, data curation. **Serieux Cyubahiro**: writing–original draft, writing–review and editing, software, formal analysis, data curation. **Ivan Steve Rwema**: writing–original draft, writing–review and editing, formal analysis, data curation. **Daniel Ukwishatse**: writing–original draft, writing–review and editing, methodology, formal analysis, data curation. **Patrick Karakwende**: writing–original draft, writing–review and editing, formal analysis. **Jean Muhire**: writing–original draft, writing–review and editing, formal analysis, data curation. **Adolphe Ndikubwimana**: writing–original draft, writing–review and editing, validation, resources. **Theoneste Ntakirutimana**: writing–original draft, writing–review and editing, formal analysis, data curation. **Pierre Dukuziyaturemye**: writing–original draft, writing–review and editing, formal analysis, software, data curation. **Didas Mugisha**: writing–original draft, writing–review and editing, formal analysis. **Canisius Gasana**: writing–original draft, writing–review and editing, formal analysis. **Edith Musabwa**: writing–original draft, writing–review and editing, formal analysis. **Felicien Irafasha**: writing–original draft, writing–review and editing, formal analysis. **Celestin Banamwana**: writing–original draft, writing–review and editing, formal analysis. **Frank Gasana**: writing–original draft, writing–review and editing, formal analysis. **Idrissa Nkurunziza**: writing–original draft, writing–review and editing, formal analysis, supervision, methodology, validation, software. **Felix Kitema Rubuga**: writing–review and editing, writing–original draft, methodology, supervision. **Deborah Oluwaseun Shomuyiwa**: writing–original draft, writing–review and editing, formal analysis, software, data curation. **Don Eliseo Lucero‐Prisno III**: writing–original draft, writing–review and editing, data curation, validation.

## Ethics Statement

Prior to initiating the research, we obtained ethical clearance (CMHS/IRB/004/2023) from the Institutional Review Board (IRB) of the University of Rwanda, College of Medicine, and Health Sciences. After receiving IRB Ethical clearance, we requested permission from the University of Rwanda to conduct data collection. All participants received a comprehensive briefing about the study's nature, objectives, and significance prior to completing the questionnaire. Students who were eligible to participate in the IRB‐approved study had to provide informed consent by agreeing to the terms of privacy and confidentiality outlined in the IRB‐approved consent form. Participation in the study was entirely voluntary, and participants had the right to withdraw at any time. We guaranteed participants that their information would be kept confidential and their identity would not be revealed during data analysis and reporting, as stipulated in the consent form.

## Conflicts of Interest

Deborah Shomuyiwa is an Editorial Board member of Public Health Challenges and a co‐author of this article. Don Eliseo Lucero‐Prisno is Editor‐in‐Chief of the journal and co‐author of this article. They were excluded from the peer‐review process and all editorial decisions related to the acceptance and publication of this article.

## Data Availability

The data that support the findings of this study are available from the authors, but restrictions apply to the availability of these data, and they are not publicly available. Data are, however, available from the authors upon reasonable request.
